# Impact of TNF inhibitors on inflammation-associated cognitive dysfunction in patients with rheumatoid arthritis: a prospective analysis

**DOI:** 10.3389/fmed.2025.1561140

**Published:** 2025-04-11

**Authors:** Natalia Mena-Vázquez, Fernando Ortiz-Márquez, Teresa Ramirez-García, Celia Gillis-Onieva, Pablo Cabezudo-García, Aimara García-Studer, Arkaitz Mucientes, Jose Manuel Lisbona-Montañez, Paula Borregón-Garrido, Patricia Ruiz-Limón, Sara Manrique-Arija, Laura Cano-García, Pedro Jesús Serrano-Castro, Antonio Fernández-Nebro

**Affiliations:** ^1^The Biomedical Research Institute of Malaga and Platform in Nanomedicine (IBIMA BIONAND Platform), Málaga, Spain; ^2^UGC de Reumatología, Hospital Regional Universitario de Málaga, Málaga, Spain; ^3^Departamento de Medicina, Universidad de Málaga, Málaga, Spain; ^4^UGC de Neurociencias, Hospital Regional Universitario de Málaga, Málaga, Spain; ^5^Unidad de Gestión Clínica de Endocrinología y Nutrición, Hospital Universitario Virgen de la Victoria, Málaga, Spain; ^6^CIBER Fisiopatología de la Obesidad y Nutrición (CIBEROBN), Instituto de Salud Carlos III, Madrid, Spain

**Keywords:** rheumatoid arthritis, anti-TNF therapy, cognitive function, inflammation, disease activity

## Abstract

**Objectives:**

To evaluate cognitive improvement in patients with rheumatoid arthritis (RA) after 6 months of treatment with tumor necrosis factor (TNF) inhibitors, analyze associated factors, and determine the percentage of patients achieving cognitive improvement.

**Methods:**

This was a single-center prospective observational study conducted over 12 months on 70 RA patients initiating their first biologic disease-modifying antirheumatic drug (bDMARD) with a TNF inhibitor. Cognitive function was assessed at baseline and after 6 months using validated neuropsychological tests, including the Montreal Cognitive Assessment (MoCA) for global cognitive function, the digit span forward and backward tests for attention and working memory, and the Stroop-W, Stroop-C, and Stroop-CW tests for executive function and processing speed. Patient-reported outcomes were assessed using the Hospital Anxiety and Depression Scale (HADS) and the Quality of Life-Rheumatoid Arthritis Scale-II (QOL-RA II). Clinical variables, disease activity measured by the 28-joint Disease Activity Score based on C-reactive protein (DAS28-CRP), inflammatory markers including C-reactive protein (CRP) and erythrocyte sedimentation rate (ESR), and patient-reported outcomes were recorded. Associations with average CRP and Health Assessment Questionnaire (HAQ) scores were analyzed throughout the follow-up period. Cognitive improvement was defined as a ≥20% increase in MoCA test scores. Logistic regression was performed to identify factors associated with improvement.

**Results:**

A total of 70 patients (mean age, 56.2 years; 81.4% female) were included. After 6 months, patients showed significant cognitive improvement in a validated questionnaire, namely, the Montreal Cognitive Assessment (MoCA test 23.1 ± 3.6 to 24.1 ± 3.3; *p* = 0.001), particularly in the executive and memory domains. Significant improvements were also observed in the digit span forward test (*p* = 0.003), digit span backward test (*p* = 0.021), Stroop-W test (*p* = 0.040), Stroop-C test (*p* = 0.014), and Stroop-CW test (*p* = 0.035). Improvements in the MoCA were associated with educational level (*B* = 2.628; *p* < 0.001), average CRP (*B* = −0.154; *p* = 0.002), and average HAQ (*B* = −0.303; *p* = 0.022). Similar associations were found for the other tests.

**Conclusion:**

TNF inhibitor therapy in RA patients is associated with significant cognitive improvement, particularly in executive function and memory. These findings highlight the potential cognitive benefits of effective RA treatment and underscore the importance of addressing modifiable risk factors to enhance patient quality of life.

## Introduction

Rheumatoid arthritis (RA) is a chronic systemic autoimmune disease. Clinically, it is characterized by symmetrical inflammation affecting mainly the small joints. Recent studies suggest that patients with RA are at greater risk of cognitive impairment and dementia than the general population (1). The main factors associated with cognitive dysfunction in RA include chronic inflammation (2–4), age, educational level, disease duration, depression, anxiety, and pharmacological treatment (5–9).

The mechanism underlying the more pronounced cognitive impairment observed in patients with RA has also been assessed. Chronic inflammation in these patients significantly contributes to cognitive dysfunction, primarily mediated by TNF-α. Murine studies suggest that chronic inflammation alters the blood–brain barrier and damages the central nervous system (10). This observation has been extrapolated to patients with RA, where the role of TNF-α as a proinflammatory cytokine is key (2, 11). Sag et al. reported a significant decrease in levels of proteins from neurons and glial cells (markers of brain damage) in RA patients treated with TNF inhibitors (11). It has also been hypothesized that TNF-α reduces cerebral perfusion, leading to frontal and parietal hypoperfusion, which in turn causes cognitive impairment. Therefore, inhibition of TNF-α improves cognitive functioning by improving cerebral perfusion (3). Moreover, patients with Alzheimer's disease, the most common type of dementia, have high TNF-α levels (12), and perispinal etanercept (a TNF inhibitor) has been shown to rapidly improve verbal fluency and aphasia in patients with Alzheimer's disease (13).

In addition to anti-TNF-α, the effects of other types of drugs used to treat RA and cognitive impairment have also been evaluated. Glucocorticoids have been associated with memory abnormalities, whereas findings for methotrexate have been contradictory (14). In contrast, treatment with TNF inhibitors has been associated with improvements in cognitive function (3, 15, 16) and a lower risk of dementia (9, 17–19). Most studies indicate that patients treated with TNF inhibitors have a lower risk of dementia than those treated with conventional synthetic DMARDs (csDMARDs) (8, 9, 17–19).

Most studies that have evaluated the risk of cognitive impairment and dementia in patients with RA are cross-sectional or retrospective in design and are limited by the fact that they do not evaluate the effect of treatment, thus hampering our assessment of the direct role of inflammation. Moreover, most measure the presence or absence of cognitive impairment or dementia without assessing the various cognitive domains, which can be better assessed in prospective studies. Nevertheless, prospective studies to date are subject to a series of limitations: very small samples (3, 15); use of the Mini-Mental State Examination as the main tool for evaluating cognitive function [this has a smaller effect size than the Montreal Cognitive Assessment (MoCA) owing to its lower sensitivity for detecting mild cognitive deficits and the fact that they assess narrower domains] (1); and a very short period between the first and second cognitive assessment, which can generate recall bias (3).

Despite all this evidence on the contribution of inflammation to cognitive dysfunction in RA patients, primarily driven by TNF, the specific impact of TNF-α blockade on cognitive function, especially in key domains like memory and executive function, has been insufficiently explored in longitudinal studies. Therefore, the key hypothesis of the present study is that treatment with TNF inhibitors in patients with active RA not only reduces systemic inflammation but also significantly improves cognitive function over a 6-month follow-up period, especially in domains vulnerable to chronic inflammation.

The objectives of our study were as follows: (1) to describe changes in cognitive function among patients with active RA after 6 months' treatment with TNF inhibitors, using validated neuropsychological tools targeting specific cognitive domains; (2) Quantify the percentage of patients whose cognitive functions improve, worsen, and remain unchanged in different cognitive domains following treatment; (3) to analyze the correlation between clinical improvement and changes in cognitive function; and (4) to identify clinical, sociodemographic, and therapy-related factors associated with cognitive performance after treatment with TNF inhibitors. Our results could provide us with key information on the impact of TNF inhibitors on cognitive functions and their association with clinical and sociodemographic variables.

## Patients and methods

### Study design

We performed a 24-week single-center prospective observational study in a cohort of patients with established RA. The sample comprised patients with RA whose response to csDMARDs was insufficient and who had received their first biologic therapy with TNF inhibitors under conditions of daily clinical practice. The study was performed in the Rheumatology Department of Hospital Regional Universitario de Málaga (HRUM), Málaga, Spain and had been approved by the Clinical Research Ethics Committee of HRUM (code 03/2022 PI 12). All the participants gave their written informed consent before entering the study and were treated according to the ethical principles of the Declarations of Helsinki.

### Patients

RA patients fulfilling the inclusion criteria were recruited consecutively at the Rheumatology Clinic of HRUM from June 2022 to June 2023. The inclusion criteria were a diagnosis of RA based on the 2010 criteria of ACR/EULAR, age >16 years, ability to complete the study questionnaires, and a medical indication to start biologic treatment for moderate-high inflammatory activity. We excluded patients with rheumatic diseases other than RA, those with previous neurological disease unrelated to the course of RA, and patients who had previously received biologics.

### Protocol

**Figure 1:**
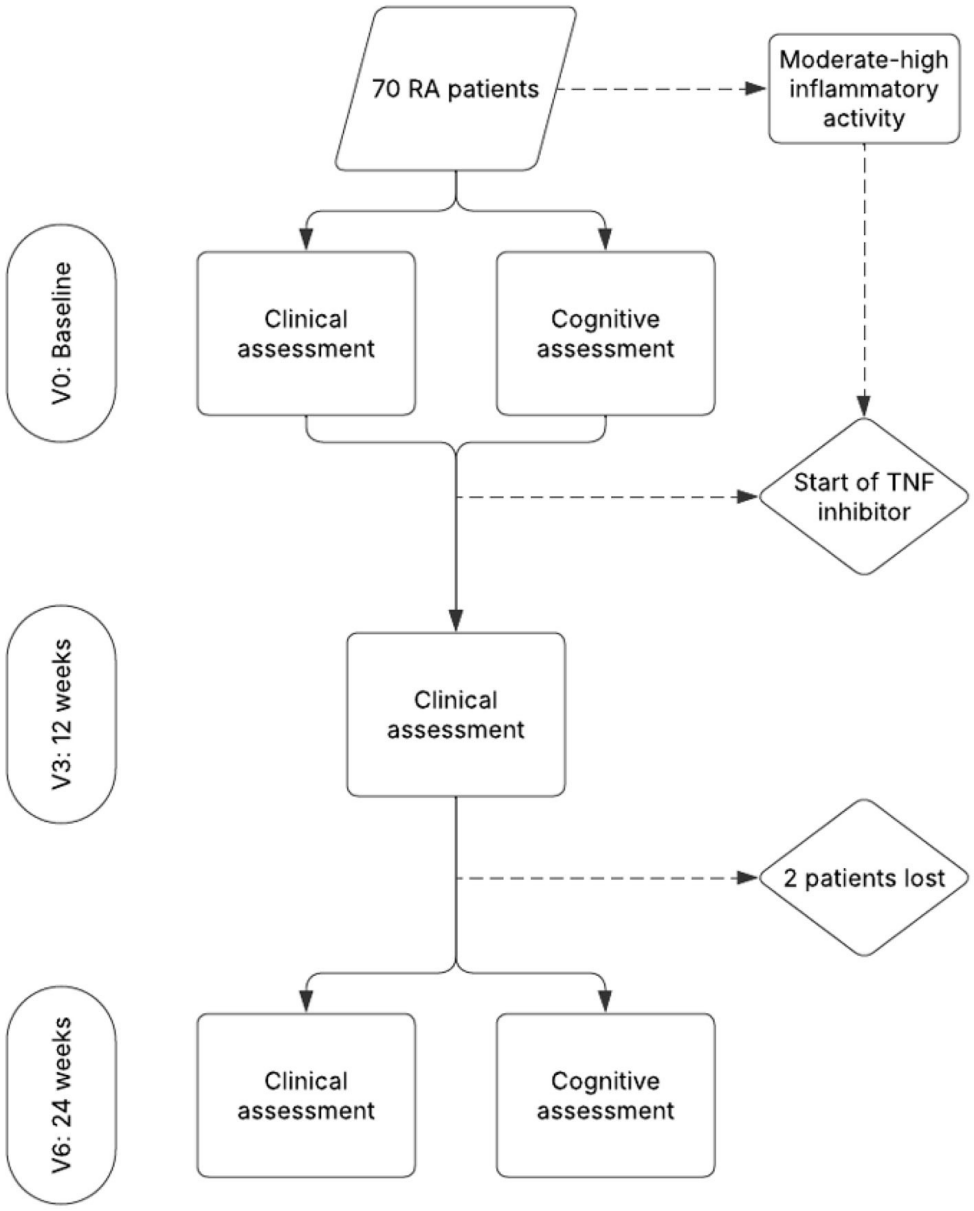
Study flow chart and patient evaluation timeline.

All the patients completed a series of neuropsychological tests at 2 timepoints: baseline (V0), that is, immediately before biologic treatment; and after 6 months of treatment (V6). They also underwent a full clinical assessment at V0, 3 months (V3), and at V6. Clinical data were collected and the physical examination performed by 2 rheumatologists; the neuropsychological tests were selected and corrected jointly by a neuropsychologist and a neurologist (Figure 1). The tests selected had been validated for different cognitive areas in RA patients. Priority was given to neurological tests that did not require manual dexterity and could be completed without time limits. These were appropriate for patients with joint deformities (4).

### Cognitive function: outcome variables

The primary outcome measure was improved performance in cognitive function according to the Montreal Cognitive Assessment (MoCA), a validated tool for assessing global cognitive function that evaluates multiple cognitive domains, including memory, attention, language, abstraction, orientation, and visuospatial-executive function, with a maximum score of 30 points. A score of <26 points was considered indicative of cognitive impairment (20). An improvement in cognitive function was defined as a significant increase in the MoCA score after 6 months of treatment. We also analyzed the change in the percentage of patients who reached a score >26 points at the end of the study period.

In addition to the MoCA, specific neuropsychological tests were used to assess different cognitive domains. Executive functions, including working memory, selective attention, and inhibition, were evaluated using the digit span backward, Stroop-C, and Stroop-CW tests, respectively (21, 22). The digit span forward test was used to assess attention and short-term memory, where participants were asked to repeat sequences of digits in the same order as presented (21). The Stroop-W test measured processing speed, requiring participants to read color words printed in black ink, while the Stroop-C and Stroop-CW tests involved naming the color of ink in color blocks or incongruent color words to assess attention and cognitive flexibility (22).

Psychiatric symptoms were evaluated using the Hospital Anxiety and Depression Scale (HADS), which consists of 14 items divided into two subscales for anxiety and depression, with scores ranging from 0 to 21 for each subscale. A score of <11 on each subscale was considered normal, while higher scores indicated the presence of anxiety or depressive symptoms (23).

Quality of life was assessed using the Quality of Life-Rheumatoid Arthritis Scale-II (QOL-RA II), a tool specifically designed for patients with rheumatoid arthritis. This scale comprises eight items that measure physical function, emotional wellbeing, social interaction, and pain, with each item scored on a scale from 0 to 10. Higher scores reflect a better quality of life (24).

### Independent clinical-epidemiological variables

As additional variables, we included epidemiological data, cardiovascular risk factors, disease characteristics, and previous treatments. The data collected at baseline were sex (male or female), ethnic group (White or non-White), age (based on date of birth), and educational level (none, primary, secondary, university). Cardiovascular risk factors were recorded at baseline and at 6 months and included smoking (active, exsmoker, non-smoker), alcohol consumption (yes/no), arterial hypertension (defined as blood pressure ≥140/90), obesity (body mass index >30), diabetes mellitus, dyslipidemia (defined as total cholesterol >200 mg/dL, low-density lipoproteins >115 mg/dL, triglycerides >200 mg/dL), and history of cardiovascular disease.

At baseline, we recorded the date of symptom onset (based on the first appearance of symptoms), the date of diagnosis according to the 2010 ACR/EULAR criteria, the time since diagnosis, and the diagnostic delay (time from symptom onset to diagnosis). The remaining clinical and laboratory variables collected at V0 and at 6 months included severity-related factors, as follows: rheumatoid factor, expressed as IU/mL; anti–citrullinated peptide antibody, also expressed as IU/mL; erythrocyte sedimentation rate, in mm/h; C-reactive protein (CRP), in mg/L; and the interleukins IL-6 and IL-1β, in pg/mL. Disease activity was evaluated using the 28-joint Disease Activity Score (DAS28), ranging from 0 to 9.4, where high-moderate activity was defined as DAS28 ≥3.2 and low activity-remission was defined as <3.2. We also took into account the Health Assessment Questionnaire (HAQ, range 0–3) at V0 and the presence of erosions (yes/no). We recorded the average DAS28, CRP, and HAQ values, defined as the mean value for these parameters during the course of the disease. In addition, we recorded treatments such as glucocorticoids and csDMARDs (methotrexate, leflunomide, sulfasalazine, hydroxychloroquine).

### Statistical analysis

After collecting the data, we performed a descriptive analysis of the results of the different questionnaires administered, epidemiological variables, cardiovascular risk factors, clinical characteristics, and drugs received at V0 and V6. The normality of the variables was evaluated using the Kolmogorov-Smirnov test. Normally distributed quantitative variables were expressed as mean (SD); non-normally distributed variables were expressed as median (IQR). Qualitative variables were expressed as absolute numbers and percentages.

Changes in cognitive performance and in the various areas and functions were evaluated by comparing normally distributed quantitative variables at V0 and V6 using the *t-*test for paired samples. Qualitative variables were evaluated using the χ^2^ test.

Bivariate correlations were made between the cognitive test results at the end of follow-up and baseline characteristics such as age, sex, educational level, and inflammation-related factors. Multivariate linear regression models were used to identify factors associated with changes in cognitive function according to the various tests administered (MoCA, digit span forward, digit span backward, Stroop-W, Stroop-C, Stroop-CW). The independent variables in these models were the baseline characteristics that proved to be significant in the correlations or were of direct clinical interest, such as epidemiological characteristics, educational level, cardiovascular risk comorbidities, and inflammation-related factors. The sample size calculation estimated that 41 patients were needed to detect a difference of at least 1.86 points on the MMSE scale (common standard deviation of 4.02, α = 0.05, power > 0.80) (15). To ensure sufficient statistical power and account for potential dropouts, we initially included 70 patients, as previously determined in our baseline cross-sectional study for comparison with a control group (4). The statistical analyses were performed using IBM SPSS Statistics for Windows, Version 28 (IBM Corp., Armonk, NY, USA).

## Results

### Clinical and epidemiological characteristics at baseline and after 6 months of treatment with TNF inhibitors

The study population comprised 70 RA patients with moderate-high inflammatory activity who started their first biologic therapy with a TNF inhibitor. Two patients were lost during the 6-month prospective follow-up, one who died of HAV infection and another who died of cervical cancer. Table 1 shows the clinical and epidemiological characteristics at initiation of the TNF inhibitor and after 6 months of follow-up.

**Table 1:** Clinical characteristics of 70 patients with RA at baseline and after 6 months of treatment with TNF inhibitors.

**Variable**	**Baseline *n* = 70**	**6 months *n* = 68**	***p*-value**
**Epidemiological characteristics**			
Female sex, *n* (%)	57 (81.4)	55 (80.9)	1.000
Age in years, mean (SD)	56.2 (12.3)	56.7 (12.0)	0.957
White race, *n* (%)	70 (100)	68 (100)	1.000
Educational level:			1.000
Basic, *n* (%)	20 (28.6)	19 (27.9)	
Higher, non-university, *n* (%)	34 (48.6)	33 (48.5)	
University, *n* (%)	16 (22.9)	16 (23.5)	
**Clinical characteristics**			
Dyslipidemia, *n* (%)	16 (22.9)	15 (22.1)	1.000
Arterial hypertension, *n* (%)	20 (28.6)	17 (25.0)	1.000
Smoking			1.000
Non-smoker, *n* (%)	31 (44.3)	30 (44.1)	
Ex-smoker, *n* (%)	21 (30.0)	20 (29.4)	
Smoker, *n* (%)	18 (25.7)	18 (26.5)	
Obesity, *n* (%)	20 (28.6)	19 (27.9)	
Diabetes mellitus, *n* (%)	8 (11.4)	8 (11.8)	1.000
Disease duration, median (IQR), months	126.4 (34.6–184.8)		
Diagnostic delay, median (IQR) months	10.5 (3.9–11.52)		
Erosions, *n* (%)	35 (50.0)	33 (48.5)	1.000
RF-positive (>10 U/mL), *n* (%)	60 (85.7)	59 (86.8)	1.000
ACPA-positive (>20 U/mL), *n* (%)	56 (80.0)	55 (80.9)	1.000
High ACPA >340 U/mL, *n* (%)	21 (30.0)	21 (30.9)	1.000
DAS28-CRP, mean (SD)	4.9 (1.15)	2.7 (1.1)	<0.001
HAQ, mean (SD)	1.4 (0.7)	0.9 (0.6)	<0.001
CRP, mg/L, mean (SD)	14.7 (10.7)	10.9 (26.3)	<0.001
IL-6, pg/mL, median (IQR)	5.4 (2.2–13.3)	2.2 (1.0–6.4)	<0.001
IL-1β, pg/mL, median (IQR)	8.2 (2.9–13.2)	3.1 (0.8–9.1)	<0.001
Erythrocyte sedimentation rate mm/h, mean (SD)	27.2 (18.0)	24.4 (19.2)	0.098
**Treatment**			
Synthetic DMARDs, *n* (%)	70 (100.0)	60 (88.2)	0.331
Methotrexate, *n* (%)	45 (64.3)	39 (57.4)	0.167
Hydroxychloroquine, *n* (%)	11 (15.7)	5 (7.4)	0.058
Leflunomide, *n* (%)	11 (15.7)	7 (10.3)	0.083
Sulfasalazine, *n* (%)	19 (27.1)	9 (13.2)	0.006
Corticosteroids, median (IQR)	5.0 (0.0–7.5)		
Corticosteroids, *n* (%)	52 (74.3)		

ACPA, anti–citrullinated peptide antibody; DAS28, 28-joint Disease Activity Score; SD, standard deviation; IQR, interquartile range; DMARD, disease-modifying antirheumatic drug; RF, rheumatoid factor; HAQ, Health Assessment Questionnaire; CRP, C-reactive protein; IL, interleukin.

Most patients were women (81.4%), and the mean age was 56.2 years. All the patients were White, and no significant changes were observed in educational level after 6 months. No relevant changes were observed for comorbid conditions such as dyslipidemia, arterial hypertension, smoking, and diabetes mellitus, or for clinical characteristics, such as erosions (*p* = 1.000), rheumatoid factor (*p* = 1.000), and anti–citrullinated peptide antibody (*p* = 1.000). However, significant improvements were observed in mean (SD) values for disease activity according to the DAS28-CRP [4.9 (1.1) vs. 2.7 (1.1); *p* < 0.001] and physical function according to the HAQ [1.4 (0.7) vs. 0.9 (0.6); *p* < 0.001]. In addition, significant reductions were observed for serum CRP (*p* < 0.001), IL-6 (*p* < 0.001), and IL-1β (*p* < 0.001).

As for treatment, all patients were receiving csDMARDs at baseline, although these were used slightly less frequently at 6 months (100 vs. 88.2%). The most significant reduction was observed for sulfasalazine (27.1 vs. 13.2%, *p* = 0.006). In contrast, no statistically significant differences were observed for methotrexate or the other csDMARDs.

### Changes in cognitive function after 6 months of treatment with TNF inhibitors

Table 2 shows the results of the cognitive tests and for anxiety and depression in patients with RA. After 6 months' treatment with TNF inhibitors, significant improvements were observed in the MoCA score (23.1 ± 3.6 to 24.1 ± 3.3; *p* = 0.001). However, the percentage of patients with cognitive impairment (MoCA < 26) did not change significantly (60.0 vs. 54.4%; *p* = 0.321).

**Table 2:** Cognitive test results and anxiety and depression at baseline and after 6 months of treatment with TNF inhibitors.

**Variable**	**Baseline *n* = 70**	**6 months *n* = 68**	***p*-value**
MoCA score, mean (SD)	23.1 (3.6)	24.1 (3.3)	0.001
Cognitive impairment (<26 MoCA), *n* (%)	42 (60.0)	37 (54.4)	0.321
Visuospatial, median (IQR)	4.0 (2.7–5.0)	4.0 (3.0–5.0)	0.135
Visuospatial successful (5 p), *n* (%)	23 (32.9)	25 (36.8)	0.621
Identification, median (IQR)	3.0 (3.0–3.0)	3.0 (3.0–3.0)	1.000
Identification successful (3 p), *n* (%)	66 (94.3)	62 (91.2)	0.182
Memory successful, *n* (%)	56 (80.0)	64 (94.1)	0.002
Attention, median (IQR)	4.0 (3.0–5.0)	4.5 (4.0–6.0)	0.009
Attention successful (6 p), *n* (%)	20 (28.6)	21 (30.9)	0.784
Verbal fluency, median (IQR)	2.0 (1.0–3.0)	2.0 (1.0–3.0)	0.311
Verbal fluency successful (3 p), *n* (%)	20 (28.6)	24 (35.3)	0.208
Abstraction, median (IQR)	1.5 (1.0–2.0)	2.0 (1.0–2.0)	0.134
Abstraction successful (2 p), *n* (%)	39 (55.7)	42 (61.8)	0.370
Delayed recall, median (IQR)	2.0 (1.0–3.0)	2.5 (1.0–3.0)	0.918
Delayed recall successful (5 p), *n* (%)	14 (20.0)	10 (14.7)	0.208
Orientation, median (IQR)	6.0 (6.0–6.0)	6.0 (6.0–6.0)	1.000
Orientation successful (6 p), *n* (%)	70 (100.0)	68 (100)	1.000
Digit span forward, mean (SD)	5.9 (2.0)	6.6 (2.0)	0.003
Digit span backward, mean (SD)	4.0 (1.7)	4.6 (1.9)	0.021
**Stroop test**			
Stroop-W, mean (SD)	93.5 (28.3)	99.7 (28.8)	0.040
Stroop-C, mean (SD)	74.7 (22.5)	80.3 (22.9)	0.014
Stroop-CS, mean (SD)	47.1 (19.0)	51.7 (20.5)	0.035
Depression HADS, mean (SD)	5.5 (3.3)	4.5 (2.8)	0.007
Depression (HADS > 11), *n* (%)	9 (12.9)	3 (4.4)	0.024
Anxiety HADS, mean (SD)	7.7 (4.2)	6.3 (3.4)	0.008
Anxiety (HADS > 11), *n* (%)	15 (21.4)	10 (14.7)	0.058
QOL-RA-1, median (IQR)	5.0 (3.0–6.0)	6.0 (5.0–8.0)	0.001
QOL-RA-2, median (IQR)	6.0 (4.0–8.0)	7.5 (6.0–9.0)	<0.001
QOL-RA-3, median (IQR)	4.0 (3.0–6.0)	6.0 (3.7–7.0)	0.005
QOL-RA-4, median (IQR)	4.0 (3.0–6.0)	6.0 (4.0–8.0)	<0.001
QOL-RA-5, median (IQR)	5.0 (3.0–6.0)	6.0 (5.0–8.0)	<0.001
QOL-RA-6, median (IQR)	4.0 (3.0–6.0)	5.5 (3.7–7.2)	<0.001
QOL-RA-7, median (IQR)	6.0 (3.0–7.5)	7.5 (6.0–9.0)	<0.001
QOL-RA-8, median (IQR)	5.0 (3.0–7.0)	7 (5.0–8.0)	<0.001

SD, standard deviation; MoCA, Montreal Cognitive Assessment; IQR, interquartile range; Stroop-W, processing speed; Stroop-C, selective attention; Stroop-CS, inhibition; HADS, Hospital Anxiety and Depression Scale; QOL-RA, Quality of Life-Rheumatoid Arthritis.

As for the specific items in the MoCA, significant increases were observed in the scores for memory function (*p* = 0.002) and attention (*p* = 0.009). However, no relevant changes were observed in the other items, such as visuospatial, identification, language, and abstraction. Significant improvements were observed in the digit span forward and backward tests (*p* = 0.003 and *p* = 0.021, respectively), as in the Stroop subtests, namely, processing speed (*p* = 0.040), selective attention (*p* = 0.014), and inhibition (*p* = 0.035).

In mental health, the mean scores for anxiety and depression according to the HADS decreased significantly (*p* = 0.007 and *p* = 0.008, respectively), as did the percentage of patients with moderate-severe depression (HADS > 11: 12.9 vs. 4.4%; *p* = 0.024). Moreover, patients reported significant improvements in quality of life (QOL-RA) in all the dimensions evaluated (*p* < 0.001 in most items).

### Factors correlated with cognitive function

At baseline and at 6 months, we evaluated the correlations between clinical and laboratory variables and the scores on the MoCA, digit span forward and backward tests, and the Stroop test (parts W, C, and CW).

Disease duration correlated negatively with Stroop-W (r = −0.292; *p* = 0.036) and Stroop-C (r = −0.365; *p* = 0.008), whereas age correlated negatively with Stroop-C (r = −0.315; *p* < 0.05), suggesting a mild-moderate impact of disease duration on cognitive performance.

A significant negative correlation was also recorded between inflammatory activity, as evaluated using the DAS28-CRP, and the MoCA at 6 months (*r* = −0.231; *p* = 0.045), suggesting that greater disease activity at baseline could be associated with impaired global cognitive performance. Similarly, we observed a negative correlation between average CRP levels and the MoCA (*r* = −0.243; *p* = 0.042) and the digit span backward test (r = −0.286; *p* = 0.023).

Negative correlations were recorded between physical function, measured using the HAQ, and most of the cognitive questionnaires evaluated: MoCA (r = −0.449; *p* < 0.001), digit span backward (*r* = −0.255; *p* = 0.049), Stroop-W (*r* = −0.288; *p* = 0.040), Stroop-C (*r* = −0.424; *p* < 0.001), and Stroop-CW (*r* = −0.432; *p* < 0.001).

Significant correlations were observed between the cognitive tests applied (Table 3). In particular, a significant correlation was observed between MoCA and the digit span forward test (*r* = 0.411), digit span backward test (*r* = 0.605), and all the parts of the Stroop test, namely, Stroop-W, Stroop-C, and Stroop CW (*r* > 0.600; *p* < 0.001 in all cases). Similarly, negative correlations were observed for levels of depression, measured using HADS, with both MoCA (*r* = −0.298; *p* = 0.022) and digit span backward (*r* = −0.287; *p* = 0.023).

**Table 3:** Correlations between baseline characteristics and cognitive function after 6 months of treatment with TNF inhibitors.

**Variable**	**MoCA V6 *Pearson p***	**Digit span forward V6 *Pearson p***	**Digit span backward V6 *Pearson p***	**Stroop-W V6 *Pearson p***	**Stroop-C V6 *Pearson p***	**Stroop-CW V6 *Pearson p***
Age, years	−0.151	−0.087	−0.095	−0.137	−0.315^*^	−0.385^*^
Body mass index	−0.028	−0.137	−0.021	−0.152	−0.251	−0.262
Disease duration	−0.196	−0.052	−0.052	−0.292^*^	−0.365^**^	−0.359
Diagnostic delay	0.034	−0.122	−0.122	0.176	0.141	0.016
DAS28-CRP	−0.231^*^	−0.159	−0.159	−0.094	−0.204	−0.181
HAQ	−0.449^**^	−0.136	−0.255^*^	−0.288^*^	−0.288^*^	−0.432^**^
C-reactive protein mg/L	−0.200	−0.074	−0.213	0.005	0.005	−0.154
Average C-reactive protein, mg/L	−0.243^*^	−0.106	−0.286^*^	−0.050	−0.148	−0.143
IL-6, pg/mL	−0.174	−0.027	−0.156	0.018	−0.018	−0.062
Erythrocyte sedimentation rate	−0.147	−0.030	−0.124	−0.025	−0.025	−0.202
MoCA	-	0.411^**^	0.605^**^	0.634^**^	0.605^**^	0.628^**^
Digit span forward	0.411^**^	-	0.522^**^	0.484^**^	0.486^**^	0.231
Digit span backward	0.605^**^	0.522^**^	-	0.394^**^	0.402^**^	0.413^**^
Stroop-W	0.634^**^	0.484^**^	0.394^*^	-	0.882^**^	0.536^**^
Stroop-C	0.670^**^	0.486^**^	0.402^**^	0.882^**^	-	0.722^**^
Stroop-CW	0.628^**^	0.231^*^	0.415^**^	0.536^**^	0.722^**^	-
HADS depression	−0.298^*^	−0.081	−0.287^*^	−0.058	−0.205	−0.205
HADS anxiety	0.203	−0.009	−0.231^*^	−0.049	−0.215	−0.215

DAS28, 28-joint Disease Activity Score; RF, rheumatoid factor; HADS, Hospital Anxiety and Depression Scale; HAQ, Health Assessment Questionnaire; IL, interleukin; MoCA, Montreal Cognitive Assessment; Stroop-W, processing speed; Stroop-C, selective attention; Stroop-CW, inhibition. ^*^p < 0.05 and ^**^p < 0.001.

### Multivariate analysis

Tables 4–6 show the results of several multivariate multiple linear regression models. The models explore the effect of various factors at baseline (V0) on cognitive performance after 6 months of treatment with TNF inhibitors (V6), according to the following: (1) MoCA, (2) digit span forward and backward, and (3) Stroop-W, Stroop-C, and Stroop-CW.

**Table 4:** Multivariate analysis of baseline characteristics associated with MoCA in RA after 6 months of treatment with TNF inhibitors.

**Baseline characteristics: MoCA**			
**Variable**	**Univariate B (95%CI)**	**Multivariate B (95%CI)**	***p*** **-value**
Age, years	−0.065 (−0.142, 0.012)		
Female sex	−0.090 (−2.523, 2.343)		
Educational level^*^	2.306 (1.117, 3.495)	2.628 (1.457, 3.799)	<0.001
Obesity (BMI≥30)	−1.069 (−3.130, 0.992)		
Arterial hypertension	−2.208 (−4.328, −0.088)		
Dyslipidemia	−1.131 (−3.474, 1.212)		
Depression (HADS)	−0.192 (−0.489, 0.105)		
Average DAS28	−0.545 (−1.515, 0.425)		
HAQ	−1.469 (−2.834, −0.104)	−0.303 (−1.009, 0.428)	0.022
Average CRP	−0.126 (−0.222, −0.030)	−0.154 (−0.248, −0.060)	0.002

^*^Educational level: secondary or university vs. primary.

The variables included in the equation were age, sex, educational level, obesity, average DAS28, and average C-reactive protein.

DAS28, 28-joint Disease Activity Score; HADS, Hospital Anxiety and Depression Scale; HAQ, Health Assessment Questionnaire; BMI, body mass index; MoCA, Montreal Cognitive Assessment; CRP, C-reactive protein.

**Table 5:** Multivariate analysis of baseline characteristics associated with digit span forward and digit span backward after 6 months of treatment with TNF inhibitors.

**Variable**	**Univariate B (95%CI)**	**Multivariate B (95%CI)**	***p*-value**
**Baseline characteristics associated with digit span forward**			
Age, years	−0.015 (−0.057, 0.028)		
Female sex	0.333 (−0.986, 1.653)		
Educational level^*^	0.390 (−0.322, 1.102)	0.847 (0.071, 1.622)	0.033
Obesity (BMI ≥ 30)	−0.922 (−2.047, 0.203)	−1.244 (−2.452, −0.036)	0.044
Arterial hypertension	−0.641 (−1.822, 0.540)		
Dyslipidemia	0.791 (−0.476, 2.058)		
Depression (HADS)	−0.042 (−0.207, 0.123)		
Average DAS28	−0.214 (−0.766, 0.338)		
HAQ	−0.413 (−1.194, 0.368)		
Average CRP	−0.123 (−0.179, −0.033)	−0.064 (−0.129, −0.010)	0.048
**Baseline characteristics associated with digit span backward**			
Age, years	−0.015 (−0.054, 0.025)		
Female sex	0.539 (−0.685, 1.764)		
Educational level^*^	0.481 (−0.178, 1.140)	0.691 (0.006,1.377)	0.048
Obesity (BMI ≥ 30)	−0.533 (−1.596, 0.529)		
Arterial hypertension	−0.306 (−1.415, 0.803)		
Dyslipidemia	0.403 (−0.788, 1.594)		
Depression (HADS)	−0.103 (−0.255, 0.048)		
Average DAS28	−0.425 (−0.922, 0.071)		
HAQ	−0.709 (−1.416, −0.002)		
Average CRP	−0.059 (−0.109, −0.008)	−0.067 (−0.124, −0.011)	0.021

^*^Educational level: secondary or university vs. primary.

DAS28, 28-joint Disease Activity Score; HADS, Hospital Anxiety and Depression Scale; HAQ, Health Assessment Questionnaire; BMI, body mass index; CRP, C-reactive protein.

**Table 6:** Multivariate analysis of baseline characteristics associated with Stroop-W, Stroop-C, and Stroop-CW in RA after 6 months' treatment with TNF inhibitors.

**Variable**	**Univariate B (95%CI)**	**Multivariate B (95%CI)**	***p*-value**
**Baseline characteristics associated with Stroop-W**			
Age, years	−0.312 (−0.945, 0.322)		
Female sex	−0.273 (−21.687, 21.142)		
Educational level^*^	14.722 (3.766, 25.678)	16.395 (3.771, 20.019)	0.012
Obesity (BMI ≥ 30)	−6.196 (−24.350, 11.958)		
Arterial hypertension	−13.670 (−31.498, 4.158)		
Dyslipidemia	−14.316 (−35.349, 6.717)		
Depression (HADS)	0.666 (−1.939, 3.272)		
Average DAS28	−2.472 (−11.353, 6.410)		
HAQ	−12.401 (−24.238, −0.564)		
Average CRP	−0.188 (−1.254, 0.877)		
**Baseline characteristics associated with Stroop-C**			
Age, years	−0.608 (−1.089, −0.127)		
Female sex	0.822 (−16.355, 18.000)		
Educational level^*^	12.877 (4.173, 20.581)	10.920 (11.301, 20.540)	0.027
Obesity (BMI ≥ 30)	−8.350 (−22.773, 6.073)		
Arterial hypertension	−18.579 (−31–905, −5.254)		
Dyslipidemia	−19.495 (−35.059, −3.932)		
Depression (HADS)	0.016 (−2.052, 2.085)		
Average DAS28	−2.981 (−9.916, 3.953)		
HAQ	−14.654 (−23.523, −5.785)	−11.288 (−21–854, −0.723)	0.037
Average CRP	−0.595 (−0.205, −1.389)		
**Baseline characteristics associated with Stroop-CW**			
Age, years	−0.397 (−1.061, −0.230)		
Female sex	0.550 (−19.758, 10.647)		
Educational level^*^	0.525 (8.673, 22.896)	13.962 (6.000, 21.924)	0.001
Obesity (BMI ≥ 30)	−0.222 (−23.006, 2.292)		
Arterial hypertension	−0.400 (−29.884, −6.619)		
Dyslipidemia	−0.355 (−32.355, −4,990)		
Depression (HADS)	−0.033 (−2.074, 1.658)		
Average DAS28	−0.226 (−11.050, 1.124)		
HAQ	−13.348 (−21.215, −5.481)	−10.875 (−19.620, −2.129)	0.016
Average CRP	−0.110 (−0.320, −0.012)	−0.098 (−0.220, −0.009)	0.039

^*^Educational level: secondary or university vs. primary.

The variables included in the equation were age, sex, educational level, obesity, average DAS28, and average C-reactive protein.

DAS28, 28-joint Disease Activity Score; HADS, Hospital Anxiety and Depression Scale; HAQ, Health Assessment Questionnaire; BMI, body mass index; CRP, C-reactive protein; Stroop-W, processing speed; Stroop-C, selective attention; Stroop-CW, inhibition.

All the models showed that educational level was the baseline factor (V0) that most affected cognitive performance at 6 months (V6), as follows: MoCA (B = 2.628; *p* < 0.001), digit span forward (*B* = 0.847; *p* = 0.033), digit span backward (*B* = 0.691; *p* = 0.048), Stroop-W (*B* = 16.395; *p* = 0.012), Stroop-C (*B* = 10.920; *p* = 0.027), and Stroop-CW (*B* = 13.962; *p* = 0.001).

Cumulative inflammatory activity over time to baseline (average CRP) was associated with poorer cognitive performance in MoCA (B = −0.154; *p* < 0.001), digit span forward (B = −0.064; *p* = 0.048), digit span backward (B = −0.067; *p* = 0.021), and Stroop-CW (B = −0.098; *p* = 0.039). Similar findings were reported for obesity, which was also associated with a poorer result in digit span forward (B = −1.244; *p* = 0.044), and functional disability (HAQ), which was negatively associated with the MoCA (*B* = −1.390; *p* = 0.022), Stroop-C (B = −11.288; *p* = 0.037) and Stroop-CW (B = −0.098; *p* = 0.039).

## Discussion

In this study, we evaluated clinical characteristics and cognitive function in 70 patients with RA who started their first biologic therapy with TNF inhibitors. Our results revealed significant improvements in inflammatory activity, functional capacity, and various cognitive aspects after 6 months of treatment. In particular, we observed increases in the global score of the MoCA (*p* = 0.001), with specific improvements in memory (*p* = 0.002) and attention (*p* = 0.009). These findings are consistent with those of previous studies, such as those by Raftery et al. (3) and Chen et al. (15), who also reported improvements in cognition at 3 and 6 months, respectively, in the Wechsler Adult Intelligence Scale and the Mini Mental State Examination in RA patients treated with TNF inhibitors. Moreover, a meta-analysis reinforced the hypothesis of a cognitive protective effect associated with TNF inhibitors based on a significant reduction in the risk of dementia among RA patients compared with synthetic DMARDs, which did not show this benefit (19, 25).

In our study, on the other hand, the percentage of patients with cognitive impairment (defined as a MoCA score < 26) did not change significantly at the end of follow-up (60.0 vs. 54.4%, *p* = 0.321), suggesting that while TNF inhibitors improve specific cognitive domains, they may not be sufficient to completely reduce the risk of cognitive impairment in affected patients. Of note, the short duration of follow-up (6 months) and the fact that a single drug was prescribed could account for these findings in this population. Long-term studies based on other interventions, such as cognitive training programs (26), could provide a more holistic approach.

After 6 months of treatment with TNF inhibitors, significant improvements in executive function and attention were observed according to the digital span forward and backward tests and the Stroop test. The Stroop test also revealed an increase in processing speed. While there have been no specific studies on these cognitive domains after treatment with TNF inhibitors, the improvement observed could be due to reduced inflammatory burden mediated by inhibition of TNF in the central nervous system (27, 28). Previous evidence shows that TNF and other proinflammatory interleukins negatively affect cognitive function via mechanisms such as disruption of the blood-brain barrier and a direct impact on key brain regions such as the hippocampus (29). Furthermore, diminished pain could go some way to improving concentration and processing capacity (30). This hypothesis is consistent with the simultaneous improvements in scores in the MoCA, digit span forward, and Stroop test that we observed.

As expected, a significant decrease was recorded in disease activity according to the DAS28-CRP (*p* < 0.001); this was accompanied by an improvement in functional disability (HAQ, *p* < 0.001). These findings are consistent with those of previous studies that reported the efficacy of TNF inhibitors for reducing inflammatory activity and improving physical function in RA patients (31, 32). We also observed improved values for inflammatory markers, such as CRP, IL-6, and IL-1β, thus supporting the hypothesis that suppression of inflammation plays a role in clinical improvement overall (33). In addition, we recorded a decrease in anxiety and depression scores, together with an improvement in the quality of life of individuals with RA (QOL-RA). These improvements in patients' psychological status and quality of life could be associated with diminished inflammatory burden, pain, and general malaise, in line with data from previous studies on the effects of TNF inhibitors (34).

With respect to the relationship between inflammation and cognitive impairment, our results support a significant association between inflammatory activity and cognitive performance. The multivariate analysis revealed that a greater baseline inflammatory burden according to average CRP was associated with lower scores on the MoCA, digit span backward test, and Stroop-CW test. These results reinforce the notion that systemic inflammation has a negative impact on cognition (27), probably via the effect of neurotoxic cytokines such as IL-2, IL-6, and TNF-α on the hypothalamus and hippocampus (28), as suggested by Fan et al. (27) and Basile et al. (35). Moreover, multiple cognitive tests revealed negative correlations for physical function, as measured using the HAQ, probably because this variable is considerably affected by inflammation, suggesting that both inflammation and physical impairment can negatively affect cognition. This observation is consistent with those of Shin et al. (36), who found that functional impairment measured according to the HAQ was associated with poorer performance in executive function and memory. Taken together, these findings highlight the importance of controlling inflammation, with the objective not only of relieving the physical symptoms, but also of preventing cognitive impairment.

As for the impact of sociodemographic factors, educational level was a key baseline factor, being consistently associated with better performance in all the cognitive tests, thus underlining the importance of promoting access to education as a key determinant of cognitive health (37, 38).

Our study is subject to a series of limitations. First, there was no control group with which to compare our findings. However, our primary objective was to evaluate changes in intraindividual cognitive function after initiation of the first biologic therapy prescribed to control inflammation. Second, while sufficient for detecting significant changes, the sample size may preclude the results from being generalized.

## Conclusions

In conclusion, TNF inhibitors not only reduce inflammation and improve physical function in patients with RA, but also have beneficial effects in specific cognitive domains and emotional wellbeing. These findings suggest that an integrated therapeutic strategy to control inflammation and improve physical function could significantly enhance quality of life in affected patients. Furthermore, appropriate educational level proved to be a key factor in mitigating cognitive impairment, thus underscoring the importance of addressing physical and cognitive aspects jointly in the management of RA. Consequently, it is important to perform more in-depth studies of the associations between cognitive function, sociodemographic factors, and the underlying mechanisms with the aim of optimizing the treatment and care of affected patients.

## Data Availability

The original contributions presented in the study are included in the article/supplementary material, further inquiries can be directed to the corresponding author.
